# The current issues of translating clinical xenokidney transplantation, with an emphasis on prevention of rejection

**DOI:** 10.3389/frtra.2025.1559512

**Published:** 2025-03-28

**Authors:** Jean Kwun, Joseph M. Ladowski, Annette M. Jackson, Stuart Knechtle

**Affiliations:** Duke Transplant Center, Department of Surgery, Duke University Medical Center, Durham, NC, United States

**Keywords:** xenotransplantation, xenotransplantation clinical trials, immunosuppression, nonhuman primate (NHP), pig-to-human transplantation

## 1 Advancing xenotransplantation: progress, challenges, and readiness for clinical trials

For decades xenotransplantation, the use of genetically modified pigs as organ donors, has held promise as a potential solution to the organ shortage. However, the field has recently experienced a resurgence and in the last two years witnessed several novel medical innovations—highlighted by two cases of clinical human cardiac xenografts and xenotransplantation in brain-dead human recipients (decedent model) (1–3). Undoubtedly, the success in extending xenograft survival in non-human primates (NHPs) fostered this translation. Progress in the NHP model is attributed to (1) additional genetic modifications of pig donors (4) and (2) availability of agents targeting the anti-CD40/CD154 signaling pathway (5–7). Despite the significant progress, there remains debate in the field regarding the ideal clinical pig: the inclusion of human complement regulatory proteins, anti-inflammatory proteins, and additional genetic mutations of glycans and SLAs. Still, consensus remains that the clinical pig genetic background should possess nullifying-mutations in the three enzymes responsible for creation of the three known xenoantigens (i.e., a TKO pig) (8).

Recent outcomes with this genetically edited pig donor have demonstrated impressively prolonged kidney graft survival, lasting up to 2 years (9). A pig with a similar level of genetic modification served as the heart donor for the historical first pig-to-human clinical xenotransplantation performed in 2022, resulting in 61-day patient survival (1). It is important to note that this transplant was not performed as part of a clinical trial, but rather through an emergency use of an investigational drug (eIND) for a recipient with no other medical life-prolonging options. However, this approval did not exclude donor animals that are not designated pathogen-free. Clearly, the federal regulatory officials viewed it as non-prohibitory and granted an additional eIND in September 2023. Subsequently, the second clinical cardiac xenotransplantation took place in September 2023 and resulted in a survival benefit of 40 days. Both experts and the general public accepted these outcomes as successful achievements but also highlight that the optimal pig genotype and immunosuppression regimen have not yet been identified. Given the regulatory issues with a clinical xenoheart study, it is likely that other organ systems will be looked to.

The greatest clinical need in solid organ transplantation exists for end-stage renal failure patients, for which over 90,000 await a kidney transplant in the United States alone (10). Allo-kidney transplantation offers significant benefits over dialysis, including improved patient survival and quality of life. However, there is huge gap between organ supply and demand and approximately 40% of patients on the waiting list die within 5 years (11). As suggested for decades, xenotransplantation could be a solution to this issue. Now the question is, are we ready for a large-scale clinical xenotransplant trial for patients with end-stage renal disease? While xenotransplantation presents many significant challenges, including physiological discordance, microbial safety, and regulatory aspects, our focus is on the current issues surrounding xenograft rejection.

## 2 Heart vs. kidney xenotransplantation

The most note-worthy of the recent xenotransplantation efforts is the clinical human cardiac and renal xenograft (1, 12, 13). The two cases of human cardiac xenotransplantation resulted in graft and recipient survival of 61 and 40 days. Additionally, no other option for life-prolonging medical or surgical therapy was available for these patients, and the alternative care pathway would likely have been a transition to comfort measures. The extension of their lives by month or two, with a potential of longer survival, is a commendable effort. Additionally, to evaluate the functionality and immunological response to a xenokidney in a human context, the decedent model has been employed (2, 3). In this model, brain-dead organ donors, deemed ineligible for organ donation due to trauma or physiological considerations, are utilized as recipients for xenokidneys or xenohearts (2, 3). The organs are monitored for signs of rejection and function with encouraging results to date. These studies provide reassurance regarding the low likelihood of immediate graft failure due to hyperacute rejection. However, it is worth noting that studies involving brain-dead individuals do not require approval from the FDA, but rather from local Institutional Review Boards (IRBs). Nevertheless, research involving brain-dead individuals would be encompassed within Clinical Trial Applications to FDA, akin to data obtained from non-clinical studies. Secondly, studies in brain-death individuals could be suboptimal due to injuries to vital organs, changes in hormones, metabolism, hemodynamics, and excessive inflammation, etc. (14). Therefore, the outcome from decedent models could be difficult to interpret and less informative than results of organ transplantation in living recipients.

In the case of clinical xenokidney transplantation, the comparison of outcomes must be against the medical alternative: dialysis. While allotransplantation is a preferable option to dialysis for most patients and associated with prolonged survival, improved quality of life, and decreased financial costs, it is unknown how xenotransplantation will compare to dialysis with respect to outcomes. Unfortunately, the most recent xenotransplantation results in NHPs with a TKO pig possessing human proteins show 40% graft loss due to rejection within 2–3 months (9). Even for patients experiencing a poor quality of life on dialysis, the current outcomes of xenokidney transplantation are not comparable to those of dialysis. However, unlike heart transplantation, xenokidney failure would not likely precipitate patient death as the patient could return to dialysis. Therefore, it will be crucial to discuss with potential candidates in a xenokidney clinical trial the relative risks following xenograft rejection, such as return to dialysis and subsequent allotransplantation. An important consideration is whether xenograft failure results in antibody development and sensitization against human kidneys. Given the significant homology between the pig and human MHC, xenotransplantation may further limit the patient's potential pool of allogeneic donor kidneys*.* This issue represents another of the points that should be addressed prior to a clinical xenokidney trial.

## 3 Unresolved issues in xenotransplantation

### 3.1 Lack of homogeneous outcomes in pre-clinical NHP study

Anand et al. present favorable outcomes in a pig-to-NHP xenokidney transplantation model employing a TKO pig with additional human proteins under anti-CD154mAb immunosuppression (9). As previously emphasized, the longest graft survival in this study is close to 2 years, with a mean survival time of 241.2 days (Median, 176 days). This achievement stands out significantly when compared to previous NHP xenograft survival rates using a similar donor pig genetic background. Nevertheless, it is imperative to not overlook the outcomes of animals that did not attain long-term graft survival. In the cited study, 40% (6 out of 15 animals) of NHP recipients did not survive past 2 months. The consistent occurrence of early xenograft failures in virtually all nonhuman primate xenotransplantation studies is a matter of concern, often without clearly discernible pathophysiology (15).

### 3.2 Lack of optimized immunosuppression

In the 1970s, clinical allotransplantation experienced a resurgence due to the discovery and implementation of a powerful immunosuppressive agent—cyclosporine. The addition of cyclosporine to the recipient's immunosuppressive regimen led to improved survival and graft outcomes. Unfortunately, the drawback of any immunosuppression drug is the overimmunosuppression of the recipient immune system and the resulting infections and side-effects. In xenotransplantation, the recognition that CD40/CD154 blockade could prolong xenograft survival led to the efforts to prolong survival with further immunosuppression. To this point, a myriad of agents targeting alternative mechanisms have been tried in xenotransplantation. Not surprisingly, the implemented clinical regimen includes diverse agents targeting many potentially relevant mechanisms, some perhaps redundant. While an aggressive immunosuppressive approach could facilitate long-term xenograft survival in a subset of animals, it concurrently led to a high incidence of infectious complications and recipient euthanasia not directly reported but reflected by highly censored data (15). One example of profound immunosuppression is the repeated use of rituximab to control post-xenotransplantation humoral responses (9, 16). While an excellent proof of concept approach, repeated depletion of B cells may not lend itself to safe human translation in the clinic. Overimmunosuppression puts patients at risk of irreversible infection and death, pertinent to the case of porcine CMV that likely contributed to the patient's demise in the first clinical xenoheart study (Table 1) (1). Other agents targeting more specific immune cell populations involved in the humoral response such as proteasome inhibitors or anti-CD38 mAbs may improve efficacy. However, these agents have not been tested in xenotransplantation yet. Several other pathological features are relevant to xenograft rejection besides AMR, including TMA and interstitial hemorrhage. It is challenging to determine if these features are truly “specific” for xenotransplantation, as we can observe similar injury patterns in other settings such as ABO mismatch in allotransplantation. Additionally, these features may occur secondary to AMR. Consequently, it remains unclear whether these factors alone are sufficient to reject the graft independently of AMR.

**Table 1:** The list of immunosuppressive agents used in the first human cardiac xenotransplantation.

Therapeutic approach	Target	Agent	Treatment time (POD^a^)	Dose
Induction	T cell	Thymoglobulin (anti-thymocyte globulin, ATG)	1, 2, 3	1 mg/kg, 1 mg/kg, 2 mg/kg
	B cell	Rituximab (anti-CD20mAb)	−1, 8	375 mg/m^2^
	Complement	Berinert (C1 esterase inhibitor)	−1, 0	20 Ukg
	Costimulation	KPL-404 (anti-CD40 mAb)	−1, 0	–
	Inflammation	Methylprednisolone	−1, 0	125 mg, 1,000 mg
Maintenance Immunosuppression	Costimulation	KPL-404 (anti-CD40 mAb)	–	Targeting appropriated drug level
	T and B cells	MMF	1–21, 53–60	500 mg BID
	T cells	Tacrolimus	35–	Trough 3–5 ng/ml
	Inflammation	Methylprednisolone	0, 1, 2	125 to 30 mg (taper)
Procedures	Antibody	Plasmapheresis/Plasma exchange	50, 51, 52, 53, 55 and 3 additional sessions	1–1.5 times the plasma volume
	Antibody	Intravenous immune globulin (IVIG)	43, 50	1 g/kg

^a^ POD, post-operation day. The information in the table was collected and modified from reference (1).

### 3.3 Lack of histocompatibility thresholds

Improvements in human allotransplant survival mirrored advancements in histocompatibility testing to assess immunologic risk and monitor *de novo* donor specific antibody development. Cellular flow cytometry crossmatch data has been generated in NHP studies, but thresholds correlated with early rejection phenotypes (such as antibody-mediated rejection and thrombotic microangiopathy) have not been establishment. Additionally, thorough investigations to determine the specificity of human preformed anti-pig antibodies across different xenotransplant models have not been reported. Current clinical histocompatibility thresholds in allotransplantation stratify patients according to immunological risk and inform clinical decision-making surrounding the need for induction therapy and baseline immunosuppression levels. Establishing appropriate recipient/pig-donor pairs in conjunction with optimal immunosuppression regimens in human xenotransplant trials will require adequate histocompatibility tools and thresholds.

## 4 Constructive future directions (from proof of concept to optimization phase)

The xenotransplantation field has achieved commendable success through the utilization of the NHP model. Studies to date serve as a proof-of-concept, demonstrating that pig-to-NHP or human transplantation can be achieved without hyperacute rejection and providing insights regarding the human immune response to a xenograft. However, the field must now transition into an optimization phase before larger human clinical trials can be initiated. To accomplish this, more mechanism-driven studies of rejection in xenotransplant models are imperative, with a focus on investigating therapeutics to target these pathways in combination with new or currently used genetically modified donor organs and appropriate histocompatibility testing.

## 5 Progress and innovation require risk

A prominent surgical educator of the 20th century, Dr. David Sabiston, warned his trainees against holding back surgical innovation by making rash pronouncements. This advice seems wise, and while we hold the opinions expressed above regarding the current immunologic risks of xenotransplantation, no one would be more delighted to see successful clinical implementation of xenotransplantation than us. In fact, we are working hard to evaluate and improve immunosuppression to permit safe and successful xenotransplantation. It is our humble suggestion that the group that leads the first clinical xenotransplant trial should possess the following: (1) a rigorously defined pig genome including information regarding off-target integration of any transgenes (2) a FDA-approved or clinically acceptable regimen that routinely leads to long-term xenograft survival (3) sufficient pre- and post-xenotransplant assays that replicate those utilized in clinical allotransplantation to monitor the immunologic and infectious status of the recipient and (4) consistent success in a preclinical model with a mechanistic explanation for outliers or early rejections. We expect that progress in the field may open the door to consistent long-term survival of xenografts.

## 6 Clinical xenotransplantation cases and clinical trials

It is indeed an exciting era for xenotransplantation. While the manuscript was under preparation and review, three xenokidney transplantations have been approved by FDA (compassionate use) and conducted during the processing this manuscript (17–19). So far, a total of six pig-to-human clinical xenotransplantation cases have been reported from 2022 to 2025, with an average patient age of 56.8 years (four males and two females) (Table 2). Similar to findings in nonhuman primate (NHP) models, some patients have experienced early xenograft rejection or complications (mean graft survival less than 2 months), while two patients remain ongoing with functioning graft, including one with the longest xenograft survival (∼3 months post-transplantation). Detailed information on the first three cases is now available in academic publications (1, 12, 13) while latter three cases are not available yet.

**Table 2:** The list of clinical xenotransplantation cases.

#	Tx Date	Recipient	Age at Tx	Organ	Performed by	Graft Survival	Recipient Survival	Diagnosis	Reference
1	01/07/22	David Bennett Sr.	57	Heart	U of Maryland	61	61 (03/08/22)	Heart failure	(1)
								Complications	
								Potential rejection	
2	09/20/23	Lawrence Faucette	58	Heart	U of Maryland	40	40 (09/20/23)	Rejection	(12)
3	03/16/24	Richard Slayman	62	Kidney	MGH	55	55 (05/10/24)	Complications	(13)
								Unrelated to rejection	
4	04/12/24	Lisa Pisano	54	Kidney	NYU	47 (removed)	86 (07/07/24)	Insufficient perfusion	N/A
								Potential rejection	
5	11/25/24	Towana Looney	53	Kidney	NYU	>82	N/A	Ongoing	N/A
6	01/25/25	Tim Andrews	66	Kidney	MGH	>21	N/A	Ongoing	N/A

As of February 2025, the U.S. FDA has approved two clinical trials for genetically engineered pig kidney transplantation into humans. The first trial, led by Massachusetts General Hospital (MGH) with eGenesis, involves up to three patients with end-stage kidney disease. The initial transplant in this series was already initiated (Case#6 in Table 2). The second trial, sponsored by United Therapeutics, has received FDA approval to transplant genetically modified pig kidneys into up to 50 patients. This large-scale study aims to evaluate the safety and efficacy of xenokidney transplantation as a solution to the organ shortage crisis. The progression, timing, and phenotype of rejection of these patients will provide the field with significant directions to pursue further advancements.
